# Biochemical Methods in Production of Three-Dimensional Scaffolds from Human Skin: A Window in Aesthetic Surgery

**Published:** 2018-05

**Authors:** Seyede-Sara Hashemi, Somayeh Jowkar, Mahdokht Mahmoodi, Ali Reza Rafati, Davood Mehrabani, Masoumeh Zarei, Abdolkhalegh Keshavarzi

**Affiliations:** 1 Burn and Wound Healing Research Center, Shiraz University of Medical Sciences, Shiraz, Iran;; 2 Department of Biology, Center of Taft, Payame Noor University, Taft, Iran;; 3 Division of Pharmacology and Pharmaceutical Chemistry, Sarvestan Branch, Islamic Azad University, Sarvestan, Iran;; 4 Stem Cell Technology Research Center, Shiraz University of Medical Sciences, Shiraz, Iran;; 5 Rohan Gene Cell Tech, Shiraz, Iran;; 6 Department of Obstetrics and Gynecology, Shiraz University of Medical Sciences, Shiraz, Iran

**Keywords:** Scaffold, ADM, Human skin, Aesthetic medicine

## Abstract

**BACKGROUND:**

Use of matrix-derived biologic scaffolds has become a treatment of choice in several clinical issues. This study assessed biochemical methods in production of three-dimensional scaffolds from human skin.

**METHODS:**

Human skin was prepared from circumcisions, washed in phosphate buffer saline (PBS) and kept at -20ºC until use. The skin samples underwent various methods. In group A, NaCl, Triton X100 and EDTA solution were used for removal of epidermis and was subdivided to three subgroups. The solution for removal of epidermis was similar for all subgroups, but decellularization was different. Group B was subdivided into 6 subgroups, NaCl in different concentrations was used for removal of epidermis and decellularization happened using SDS in various concentrations and different time intervals. Group C was subdivided to 3 subgroups, trypsin was used for removal of epidermis and decellularization was conducted applying NaOH or SDS. Washing was performed using only PBS. In group D, decellularization was done applying SDS. Histomorphometric study was conducted to compare the groups.

**RESULTS:**

No fibroblast was present in A2, B2, B4, and C3 subgroups after decellularization. Histological photographs from subgroups A1 to A3 revealed several cells and collagen fibers. Dense collagen fibers in pink color were noted in all subgroups; but, epidermis was absent.

**CONCLUSION:**

It was shown that 1M NaCl was the best solution for removal of epidermis, 0.5% SDS for 2 h was the most effective solution for decellularization and PBS was the best solution for washing, while the solutions are easily available and cost-effective.

## INTRODUCTION

Tissue engineering is a new approach in treatment of skin, skeleton, cartilage and many other tissue injuries, as traditional methods such as organ transplantation, beside their advantages have some limitations including immune responses, transplant rejection and waiting for a suitable donor.^1^^-^^3^ The idea of tissue engineering wasbased oncell isolationfrom a patient seeded onto a suitable carrierandgrafted for the same patient to replace the injured tissue.^3^^,^^4^ In tissue engineering, scaffolds were simulated from extracellular matrix that have been provided from resident cells in tissues and organs.^5^^,^^6^

Extracellular matrix are secreted into the surrounding medium to reach biophysical and biochemical support for the surrounding cells as a promising approach for tissue engineeringbased on theirconstituents of various bioactive molecules. Extracellular matrix scaffolds were shown to have a favorable regenerative microenvironment, promote tissue-specific remodeling, and play a role as an inductive template for repair and functional reconstruction of many tissues including skin, liver, kidney, small intestine, nerve, heart, bone, lung, and other organs.^7^

Use of matrix-derived biologic scaffolds hasbecome a treatment ofchoice in several clinicalissues, while there are several kinds of materials and methods of synthesis and development to preparea suitable scaffold.Synthetic organic andinorganic materials,and organic and inorganic materials of natural origin are four main types of biomaterials hat have been studied in-vitro and/or in-vivo as suitable scaffolds in tissue engineering.^6^^, ^^8^ Biocompatibility is one of the most important characteristics of materials considered for scaffold preparation, because of immunological barriers which may reject them. Cell adhesion, biodegradability,having sufficient space for cell adhesion, possessing a mechanically strong structure and reproducibility are other properties that scaffolds should have.^8^^,^^9^

Various three-dimensional scaffolds have been introduced for tissue engineering such as skin, bone, cartilage, ligament, etc.^9^ Acellular human skin has been reported as a biological extracellular matrix utilized for reconstruction and repair of skin injuries.^10^ Various human, porcine or bovine derived acellular dermal matrix (ADM) are available worldwide, reporting application of ADM using different buffers in different timings for acellularizasion of human skin.^11^^,^^12^ ADM assisted implant-based breast reconstruction was shown to have an increasing popularity over traditional sub muscular techniques.^13^ It has also been successfully used in reconstruction of orbital,^14^ and diaphragmatic walls,^15^ and repair of Achilles tendon,^16^ chronic diabetic foot ulcers,^17^ and hernias.^18^ In this study, we compared different methods of acellularization of human skin dermis to obtain ADM scaffolds to be inexpensive, and appropriate for seeding of mesenchymal stem cells (MSCs).

## MATERIALS AND METHODS

Human skin was prepared from circumcisions undertaken in Department of Plastic Surgery, Shiraz University of Medical Sciences, Shiraz, Iran. The tissue samples were kept in phosphate buffer saline (PBS: Sigma Aldrich, USA), while penicillin-streptomycin (Sigma Aldrich, USA) and fungisone (Sigma Aldrich, USA) were also added. After 3 times washing with PBS, subdermal fat tissue and hair were excised and the remained tissue was kept at -20ºC until use. The skin samples underwent various methods (as groups A to D) to reach an acellular dermal matrix (ADM) and summarized in Table 1.

**Table 1:** Different decellularization methods used three-dimensional scaffold from human skin

**Protocol**	**Removing epidermis**	**Decellularization**	**Washing**
A1	NaCl 1M+Triton X100 0.5%+EDTA 10mM (Shaked in incubator 37ºC-24h)	SDS 0.5%+HEPS 10mM+ EDTA 10mM (Shaked in incubator 37ºC- 1h)	Triton ×100 0.5%+EDTA 10 mM
A2	NaCl 1M+Triton X100 0.5%+EDTA 10mM (Shaked in incubator 37ºC-24h)	SDS 1%+HEPS 10mM+ EDTA 10mM (Shaked in incubator 37ºC- 1h)	Triton ×100 0.5%+EDTA 10mM
A3	NaCl 1M+Triton X100 0.5%+EDTA 10mM (Shaked in incubator 37ºC-24h)	SDS 2%+HEPS 10mM+ EDTA 10mM (Shaked in incubator 37ºC- 1h)	Triton ×100 0.5%+EDTA 10mM
B1	NaCl 1M (Shaked in incubator 37ºC-24h)	SDS 0.5% (Shaked in 25ºC- 1h)	PBS
B2	NaCl 1M (Shaked in incubator 37ºC-24h)	SDS 0.5% (Shaked in 25ºC- 2h)	PBS
B3	NaCl 1M (Shaked in incubator 37ºC- 24h)	SDS 2% (Shaked in 25ºC- 1h)	PBS
B4	NaCl 1M (Shaked in incubator 37ºC- 24h)	SDS 2%+HEPS 10mM+ EDTA 10mM (Shaked in incubator 37ºC- 2h)	Triton ×100 0.5%+EDTA 10mM
B5	NaCl 1.5M (Shaked in incubator 37ºC- 24h)	SDS 2% (Shaked in 25ºC - 1h)	PBS
B6	NaCl 2M (Shaked in incubator 37ºC- 24h)	SDS 2% (Shaked in 25ºC - 1h)	PBS
C1	Trypsin 0.25% (Shaked in incubator 37ºC- 15h)	NaOH 1M (Shaked in 25ºC- 16h)	PBS
C2	Trypsin 0.25% (Shaked in incubator 37ºC- 18h)	NaOH 1M (Shaked in 25ºC- 1h)	PBS
C3	Trypsin 0.25% (Shaked in 25ºC- 12h)	SDS 0. 1% (Shaked in 25ºC- 12h)	PBS
D	(-196 ºC -48h) NaCl 1M (Shaked in incubator 37ºC- 24h)	SDS 2% (Shaked in 25ºC- 1h)	PBS

In group A, NaCl, Triton X100 and EDTA solution were used for removal of epidermis. In summary, human skin was cut into pieces of 0.5×0.5×0.3 cm^3^ using a sterile blade. Group A was later subdivided to A1–A3 subgroups based on the undertaken method. As Table 1 shows, the solution for removal of epidermis was similar for all subgroups applying 1M NaCl , 0.5% Triton X100 and 10mM EDTA solution while were shaken in incubator at 37ºC for 24 h. Also, the washing solution for all subgroups was identical using 0.5%Triton X100 and 10mMEDTA for 2 times.

Decellularization was different in all subgroups using sodium dodecyl sulfate (SDS) in concentration of 0.5% (A1), 1% (A2) and 2% (A3) while, 10mM HEPES and 10mM EDTA solutions were also added to all subgroups and kept in incubator at 37ºC for 1 h. Before being lyophilized, penicillin-streptomycin and fungisone were added to the solution. In group B which was subdivided into 6 subgroups (B1 to B6), NaCl in different concentrations (1, 1.5 and 2M) were used for removal of epidermis (Table 1). Decellularization happened using SDS in various concentrations (0.5%, 1% and 2%) and in different time intervals. Washing was undertaken applying mostly PBS, while in one case, 0.5% Triton X100 and 10mM EDTA were used.

In group C which was subdivided to 3 subgroups (C1 to C3), 0.25% trypsin in different time intervals (15, 12, and 18 h) was used for removal of epidermis (Table 1). Decellularization was conducted applying 1M NaOH or 0.1% SDS in different time intervals (1, 12, 16 h). Washing was performed using only PBS. In group D before removal of epidermis, the tissue sample was kept in -196ºC for 48 h and later, 1M NaCl was added. Decellularization was done applying 2% SDS in time interval of 1 h. Washing was conducted by PBS. 

Histomorphometric study was undertaken before lyophilization of samples as follows: All tissue samples were fixed in 5% buffered formalin and subsequently embedded in paraffin. Tissue sections (5 microns thickness) were provided and stained with H&E, Verhoeff’s and Alcian blue techniques and visualized under Olympus BX51 microscope. SPSS software (Version 16, Chicago, IL, USA) was used for statistical analysis. One way ANOVA was used to compare the groups. A p value less than 0.05 was statistically considered significant.

## RESULTS

**Fig. 1:**
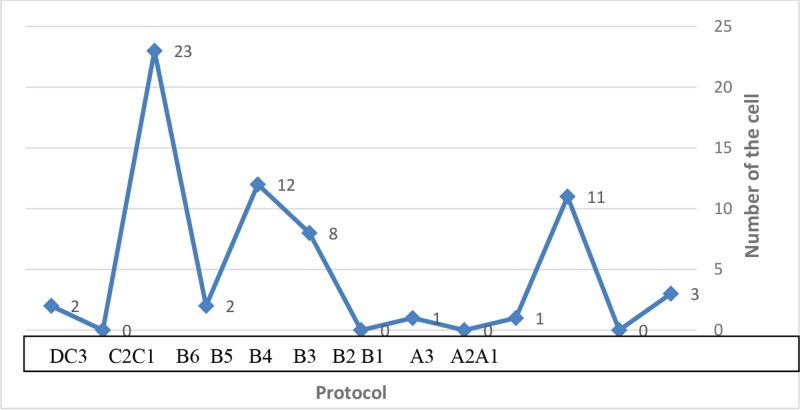
Average number of cells counted in studied groups after various staining methods.

**Fig. 2:**
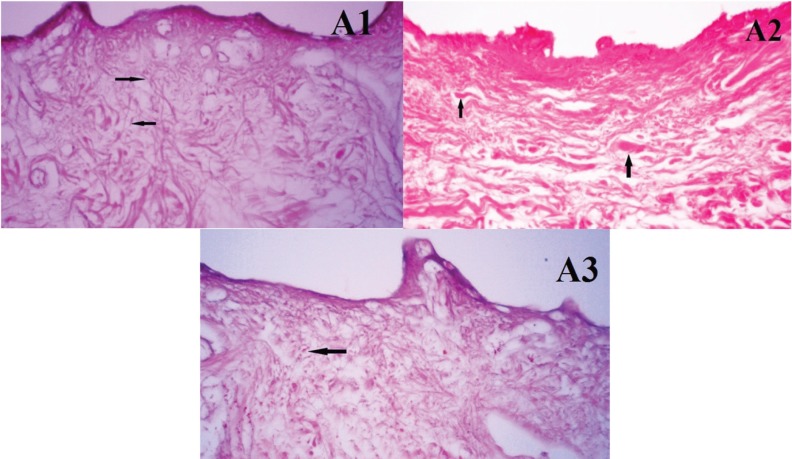
Histological photographs of ADM samples from group A. A1, A2 and A3. The number of cells was observed in four different fields in A1 and A3 (black arrow: fibroblasts or remains of them). There was no nucleus or its remains observed in A2 (black arrow: collagen fibers, ×100).

As Figure 1 shows, no fibroblast was present in A2, B2, B4, and C3 subgroups after decellularization, while in other subgroups; various numbers of fibroblasts were visible. Histological photographs of ADM samples from subgroups of A1 to A3 denoted to the number of observed cells and collagen fibers in four different fields for each sample. No fibroblast was noticed in A2, while in other subgroups, the cells were present showing a complete decellularization has been undertaken in A2 subgroups. Dense collagen fibers in pink color were also noted in all subgroups; but, epidermis was absent (Figure 2).

**Fig. 3:**
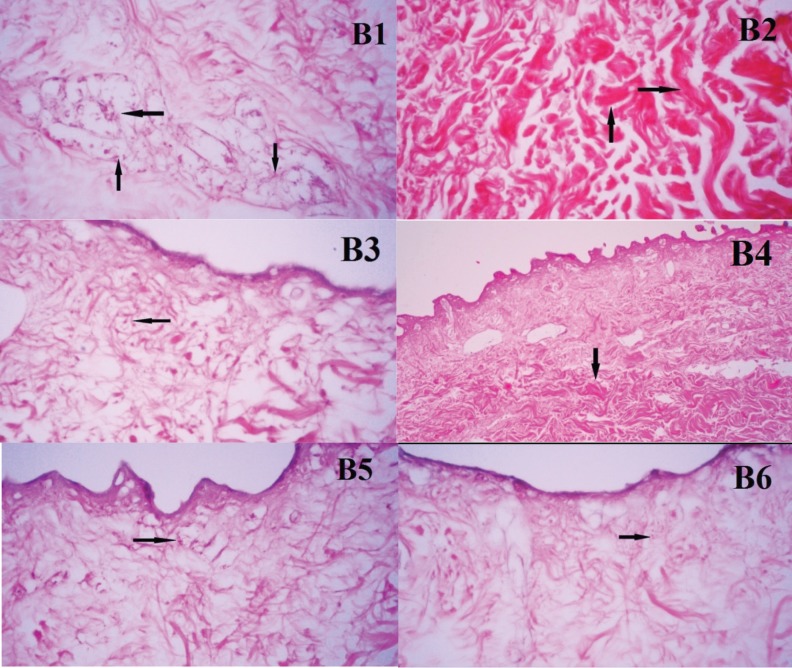
Histological photographs of ADM samples from group B. B1, B2, B3, B4, B5, and B6. The number of cells was observed in four different fields in B1, B3, B5, and B6 (black arrow: fibroblasts or remains of them). There was no nucleus or its remains observed in B2 and B4 (black arrow: collagen fibers, ×100

**Fig. 4:**
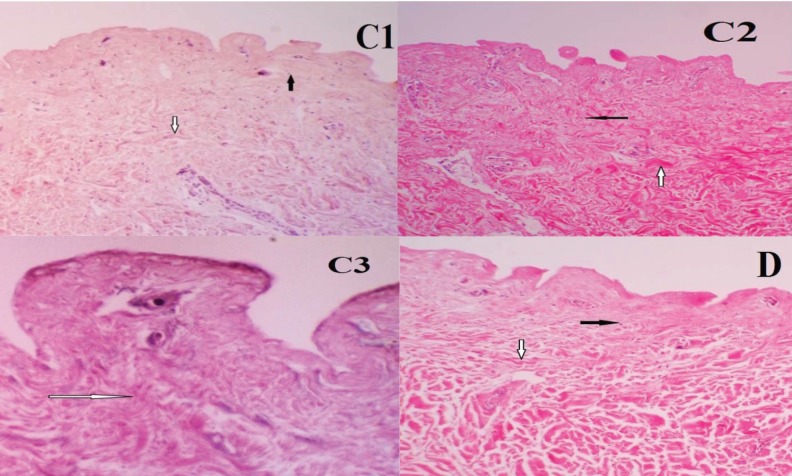
Histological photographs of ADM samples from group C. C1, C2 and C3. The number of cells was observed in four different fields in C1 and C2 (black arrow=fibroblasts or remains of them). There was no nucleus or its remains observed in C3 (black arrow=collagen fibers). Magnification=×40.Histological photographs of ADM samples from group D. The number of cells was observed in four different fields in D. (White arrow=collagen fibers, black arrow=fibroblast cell. Magnification=×100)

Figure 3 denotes to the number of observed cells and collagen fibers in four different fields of each sample. Collagen fibers in all subgroups could be seen in pink color. Fibroblasts were absent in B2 and B4 subgroups after decellularization and were visible in remained subgroups showing that the treatment time in this study (2 h) with 0.5% and 2% SDS solutions together with 10 mM HEPES and 10 mM EDTA were the most efficient methods of decellularization. In group C, collagen fibers were noticed in all subgroups. Fibroblasts were absent in C3 subgroup after decellularization and were visible in the remained subgroups showing that treatment with 0.1% SDS in 12 h time interval was the most efficient method of decellularization (Figure 4). 

**Fig. 5:**
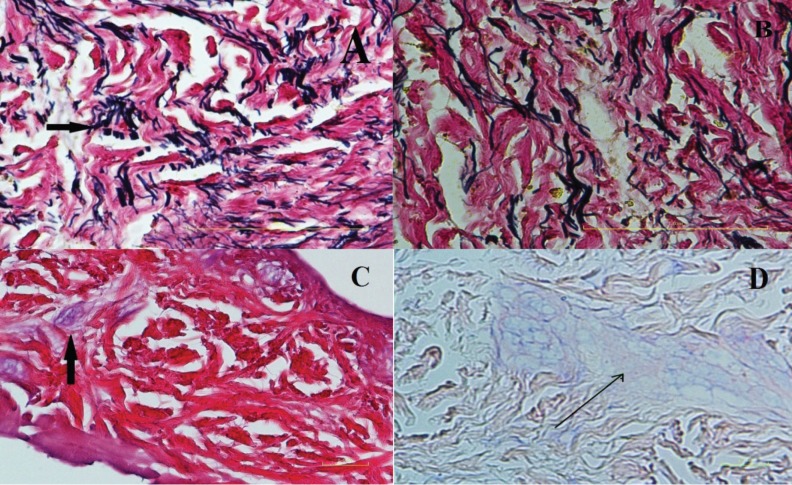
Histochemical staining of elastic fibers (ELAST) with Verhoeff staining in A (A2)- B (B2), present in NaCl-Triton- EDTA ADM, and NaCl ADM. Dark to black elastic fibers (black arrow=elastin fibers) are seen in B better than A. Magnification ×400. Histochemical staining of acid glycosaminoglycans with *Alcian blue stain* present in NaCl-Triton- EDTA ADM C(A2), and NaCl ADM D (B2). Blue areas: secreted matrix (black arrow=glycosaminoglycans). Magnification ×400

In group D, collagen fibers were detected in all subgroups. Treatment in -196ºC for 48 h and later subjecting the samples in NaCl for interval time of 24 h and decellularization with 2% SDS did not lead to a cell removal demonstrating that this method was not effective to remove the fibroblasts from tissue samples (Figure 4). Figure 5 reveals the presence of elastic fibers using Verhoff’s staining method as coarse, abundant black elastic fibers in ADM samples. Hyaluronic acid (acidic glycosaminogiycans) was evaluated by Alcian Blue staining method in the basement membrane and around the dermal vascular bundles of ADM samples (Figure 5).

## DISCUSSION

The prominent effect of extracellular matrix (ECM) scaffolds supporting tissue regeneration is dependent on their maintained 3D structure and bioactive ingredients. These decellularized matrix scaffolds could be revitalized before any grafting by addition of stem cells, fibroblasts, or keratinocytes to improve wound healing.^19^^,^^20^ These scaffolds have important role in physico-chemical process of cell growth.^21^ ECMs contain proteins such as gelatin, collagen and fibronectin providing a porous matrix that help a better adhesion and replacement of cells in the tissue. In production of scaffolds, their adverse effects such as inflammation and early degradation by enzymes should be prevented.^22^^,^^23^

Our findings demonstrated that A2, B2, B4, and C3 subgroups lacked presence of fibroblast cells after decellularization, but collagen bundles were visible in these subgroups, among them B2 and C3 had better results regarding presence of collagen and elastic fibers. It was observed that an increase in incubation time could decrease cell number of the dermal skin in B2 subgroup. Increase in de-cellularization time in B4 subgroup also lead to a complete cell removal from the tissue samples. It was shown that 1M NaCl for 24 h and 0.5% SDS for 2 h was the best method of cell removal. Chen *et al.* findings were identical to our results regarding the increase in incubation time by SDS to be effective in cell removal from the tissue samples.^24^

The effect of hypertonic and hypotonic NaCl solution in cell lysis has been previously reported.^25^^,^^26^ SDS was shown to be more effective in decellularization of tissue rather than other detergents.^27^^,^^28^ SDS was found to be effective in removal of nucleus from dense tissues such as kidney in comparison to triton 100x.^27^^,^^28^ Similar to our study, Chen and colleagues reported that using 25% trypsin for 18 h for removal of epidermis and SDS for 12 h in room temperature for cell removal and decellularization would be an efficient method.^29^ Nie *et al.* in local delivery of adipose-derived stem cells via acellular dermal matrix as a scaffold was successful and showed to accelerate the wound healing.^30^ Zhou *et al. *showed that transplantation of ADM together with bone marrow derived stem cells could partly promote the regeneration of injured anal sphincter and lessen the formation of cicatrix in experimental rat model.^31^

As the main goal of using scaffolds in aesthetic medicine is to be host compatible, biodegradable, non-immunogenic, inexpensive and promote the repair and reconstruction of injured tissues or organs, they should and have the potential to promote cell growth via biological and mechanical aspects. Here, we introduced four protocols of acellularization as the most effective methods to reach an inexpensive and efficient scaffold to help clinicians working in healing process. 1M NaCl was the best solution for removal of epidermis, 0.5% SDS for 2 h was the most effective solution for decellularization and PBS was the best solution for washing, while all of them are easily available and can be provided economically.
